# Romeo swipes Juliet left!? Problematic online dating app use and problematic pornography consumption are associated with a weaker sense of existential meaning in romantic love

**DOI:** 10.1186/s40359-026-05173-2

**Published:** 2026-07-21

**Authors:** Reza Shabahang, Zsolt Demetrovics, Mehdi Akbari, Gaëlle Ouvrein, Ágnes Zsila

**Affiliations:** 1 College of Human Sciences and Culture, Flinders University Institute for Mental Health and Wellbeing, Flinders University, Bedford Park, South Australia Australia; 2 Graduate School of Health, University of Technology Sydney, Sydney, Australia; 3 Institute of Psychology, ELTE Eötvös Loránd University, Budapest, Hungary; 4 Centre of Excellence in Responsible Gaming, University of Gibraltar, Gibraltar, Gibraltar; 5 Department of Clinical Psychology, Faculty of Psychology and Education, Kharazmi University, Tehran, Iran; 6 Faculty of Social Sciences and Solvay Business School, Vrije Universiteit Brussel, Brussels, Belgium

**Keywords:** Dating apps, Online dating, Pornography consumption, Existentialism, Love, Mindful use of social media

## Abstract

**Background:**

Although prior studies have examined the psycho-socio-sexual outcomes associated with problematic engagement with online dating apps and pornography, these behaviors remain largely unexplored from an *existential* perspective. Romantic love can also be viewed through an existential lens (e.g., contributing to one's sense of essence). This study investigated the relatively understudied relationships between problematic online dating app use, problematic pornography consumption, and existential perceptions of romantic love.

**Methods:**

A total of 664 Iranian adults (*M*_*age*_ = 26.4, *SD*_*age*_ = 7.6) who used dating apps and pornography websites were surveyed. Structural equation modeling was conducted to assess the relationships among the study variables.

**Results:**

The results showed that, controlling for demographics, problematic online dating app use and problematic pornography consumption were negatively associated with existential reflection on love and perceived meaningfulness of love. Both behaviors were positively associated with a pragmatic view of love and emotional promiscuity. A positive association was found between problematic online dating app use and problematic pornography consumption. Additionally, mindful social media use, which has recently been suggested as a protective factor among social media users, demonstrated a strong negative association with both problematic behaviors.

**Conclusion:**

Considering the potential for negative experiences associated with online dating app use (e.g., partner choice overload) and pornography consumption (e.g., sexual objectification), problematic engagement with these platforms may, to some extent, contribute to the commodification of romantic relationships and the fragmentation of existential perceptions toward romantic love. *Mindful* use of these platforms may therefore need to be considered by users.

## Introduction

### Online dating app use and pornography consumption

The use of online dating apps and engagement with pornography are widespread behaviors that, in many cases, appear to be interconnected [102]. Dating apps have experienced considerable growth in popularity, with millions of active users worldwide [98]. These platforms are frequently used by individuals seeking romantic or intimate connections and are, in some cases, displacing other traditional meeting avenues (e.g., meeting through family, meeting in social gatherings; [89]). The number of available dating platforms and their corresponding financial success have steadily increased, evidenced by millions of downloads per month and billions of dollars in revenue [98]. Likewise, pornography websites receive billions of visits each month [97], with pornography consumption being a common activity across various age groups globally [10]. In some cases, pornography consumption appears to be displacing real-life sexual encounters, as some individuals may show a preference for masturbation to pornography over partnered sex [106].

### *Frequent* and *problematic* engagement with online dating apps and pornography

Engagement with dating apps and pornography may become compulsive, with some individuals experiencing difficulties in regulating their use of these platforms and exhibiting addiction-like symptoms (e.g., spending increasing amounts of time to achieve the same level of pleasure as before). Emerging research has approached online dating app use (e.g., [43, 75]) and pornography consumption (e.g., [18]) through the framework of behavioral addiction (*Components Model of Addiction*; [44]). Although prevalence rates for problematic online dating app use remain scarce, researchers have raised concerns regarding a potential upward trend in such behaviors (see [13] for a review). In contrast, the prevalence of problematic pornography consumption has been extensively documented. For instance, Bőthe et al. [14] estimated that 3.2–16.6% of individuals across samples from 42 countries were at risk of experiencing problematic pornography consumption.

Research on the psycho-socio-sexual outcomes associated with time spent on, and problematic engagement with, online dating apps and pornography is expanding. Both being a user of online dating apps and problematic use of these platforms have been linked to dysfunctional psychological, social, and sexual outcomes. However, existing studies on online dating app use have primarily focused on usage frequency. A recent review highlighted that a significant body of research has reported negative associations between online dating app use and mental health (e.g., more symptoms of depression, anxiety, and stress; [20]). Users of online dating apps, to some extent, appear to experience negative psychological states, such as distress and sadness, compared to non-users [35, 54]. Problematic use of these platforms has been reported to be associated with psychological and social sensitivities among users, such as social interaction anxiety, rejection sensitivity, upward social comparison, and fear of being single [55, 100]. Furthermore, online dating app use has been associated with unhealthy non-sexual behaviors (e.g., disordered eating, unhealthy weight control practices; see [20] for a review) and risky sexual behaviors and choices (e.g., hypersexual tendencies and difficulties in self-regulating sex-related behaviors; [26, 36]). What is more, problematic online dating app use has been linked to other behavioral addictions, such as problematic social media use and problematic pornography consumption (e.g., [43]). Despite these findings, there remains a need for a more nuanced understanding of the outcomes associated with online dating app use, particularly from the perspective of *problematic* engagement (see [13]). This distinction is important, as a growing body of literature suggests that frequent use and problematic use of new media may have different correlates or varying degrees of association with the same cognitive, emotional, and behavioral outcomes (see *Goldilocks Hypothesis*; [82, 83]).

Regarding pornography consumption, the literature is extensive (see [104] for a review). Research has examined pornography consumption through both the lens of frequency of use and the lens of problematic use, with a growing focus on the latter due to its more significant clinical implications. For instance, Bőthe et al. [17] found that problematic high-frequency users reported higher levels of hypersexuality, basic psychological needs frustration, depression, boredom susceptibility, relatedness frustration, and competence frustration compared to non-problematic high-frequency pornography users. Studies on problematic pornography consumption have consistently identified its associations with a broad range of adverse outcomes, including stress, anxiety, depression, emotion dysregulation, loneliness, deficits in sexual perceptions and functioning, insomnia, suicidal ideation, and life dissatisfaction [6, 15, 16, 56, 72].

Despite a growing body of research examining the outcomes associated with problematic online dating app use and problematic pornography use, certain areas remain underexplored. Notably, one question has received little attention in academic discourse: *What are the existential implications linked to these problematic behaviors?* Scant scholarly attention has been devoted to investigating whether problematic engagement with these platforms is associated with users’ attitudes toward romantic love, particularly regarding the extent to which they view romantic love as existentially meaningful, post-materialist, and worthy of fidelity. Investigation into this relationship is warranted as romantic love could be perceived, understood, experienced, and studied through an existential lens (see [95]), and such problematic engagement with these platforms may be linked to altered existential views on romantic love.

### Media use and its potential existential contributions

Existentialism is a philosophical perspective that explores fundamental questions regarding purpose, meaning, values, human struggles, and the nature of existence (e.g., reflections on the self, others, the world, and life). In many respects, we interpret ourselves, others, our surroundings, and our existence through an existential lens, for instance, when confronting existential anxieties such as a heightened awareness of one's own mortality following a traumatic experience (e.g., a life-threatening illness or injury), or a loss of purpose following the loss of a loved one (see [53]).

Studying and discussing media and media users from an existential perspective and exploring the potential contribution of media use to existential and meaning-related perceptions and evaluations has garnered growing scholarly attention in recent years. According to *Media Effects Theories* (e.g., Cultivation Theory, Social Cognitive Theory of Mass Communication; see [101] for a review), media exposure and presence within mediated environment (e.g., being a social media user) has the potential to shape users' attitudes, norms, ideals, expectations, and interpretations, effects that may also extend to existential dimensions (see *Existential Media*; [66]).

Investigating media use and user-media relationships through an existential lens is particularly relevant, as human existence has undergone some transformations with the advent and pervasive presence of media, especially newer interactive media forms (e.g., artificial intelligence-powered companionship chatbots; see [25] for a discussion). Several scholarly discussions have emerged on this theme in recent years. For instance, Oliver et al. [74] proposed the concept of *Self-Transcendent Media Experiences*, arguing that positive media (e.g., hope-inspiring films, documentaries on altruistic acts, emotionally moving YouTube videos) may lead users to transcend personal concerns, recognize shared humanity, appreciate moral beauty and hope, and experience a sense of unity with humanity and nature (existential meaningfulness in general). Kruzan [64] suggested that the media, particularly interactive new platforms (e.g., social media), may alleviate existential concerns by enabling individuals to leave traces (e.g., personal images uploaded on the internet), potentially fostering a sense of symbolic immortality (a god-like existence) and transcending physical limitations, as digital traces can persist beyond one’s lifetime.

Beyond these theoretical discussions and speculations, empirical research has begun to examine the links between media use and users' existential dimensions. Shabahang et al. [90, 92] recently reported preliminary findings on the association between problematic consumption of negative-tone news (commonly referred to as doomscrolling) and existential attitudes. Their findings indicated a link between doomscrolling and heightened existential anxieties, as well as misanthropy (i.e., distrust or disdain for humanity). The authors posited that compulsive consumption of negative news (e.g., reports on wars, authoritarian regimes, genocides) may indirectly traumatize problematic consumers (*media-induced PTSD*; see [1]), potentially undermining their fundamental assumptions about a peaceful world and the inherent goodness of humankind (see *Shattered Assumption Theory*; [58]). In a study conducted during the COVID-19 pandemic, a period during which existential challenges were pronounced for many, such as a heightened awareness of mortality, [52] found that social media use was indirectly associated with greater meaning in life, and this association was explained by lower existential isolation. In another study conducted during the COVID-19 pandemic, Chen et al. [24] surveyed self-quarantined individuals and found that media involvement was positively correlated with death anxiety. Haley [50] reported that individuals who digitally disconnected during a death-thought awareness task showed higher death-related thoughts compared to those who remained connected (i.e., had access to their smartphones and could check their friends online). Etgar et al. [32] found a positive association between death anxiety and the frequency of taking and sharing selfies on social media. Alkhalifah et al. [3] recently investigated existential concerns related to artificial intelligence and reported that such concerns were prevalent among their participants. Taken together, these theoretical and empirical studies and arguments suggest that media may exert existential influences on users to some extent. The present study aims to provide preliminary insights into the existential outcomes associated with problematic engagement with online dating apps and pornography, particularly in relation to attitudes toward the existential meaning of romantic love.

### The potential existential nature of romantic love and the potential connection between romantic love and existential states

In many respects, romantic love appears to be an inherent aspect of human existence, with both love and the need to be loved regarded as fundamental to human nature. Research suggests that romantic love has the potential to enhance well-being to some extent by increasing happiness, life satisfaction, optimism, and a sense of purpose, while also mitigating psychopathological symptoms (see [41] for a review). One of the central dimensions of romantic love is its existential significance, both as a component of existential experience (e.g., contributing to self-existence and life's meaningfulness) and in its connection to existential states (e.g., existential well-being). For some individuals, romantic love can serve as a cornerstone of existence [22, 95], offering a sense of being and engagement with life and the world [23]. They may experience a feeling of strong loneliness when a meaningful romantic love is not present.

Alberoni [2] conceptualized falling in love as a form of dementia of one's prior existence, proposing that “no one can fall in love if he or she is even partly satisfied with what he or she has or what he or she is” (p. 109). Sköld and Roald [95] described romantic love as broadening one's awareness of the world and life itself, noting that “Just as depression closes the world down, love opens it up” (p. 378). Vosmer [105] characterized lovesickness (i.e., negative emotions associate with an inability to be with one’s beloved, either physically or emotionally) as “an existential issue” (p. 386). Indeed, some individuals interpret love within the broader existential framework of the self [80], and some define and encapsulate their identity through their role as a lover and their lived experiences of love [69].

Gawda and Korniluk [37], based on their findings on the association between romantic love and existential anxiety, suggested that romantic love, through its potential links to closeness and positive emotions, may serve as a protective buffer against existential vacuum (i.e., a state characterized by feelings of emptiness and an inability to find or create meaning in life). Similarly, drawing on *Terror Management Theory*, Mikulincer et al. [71] suggested that close relationships, including romantic love, could serve as a “symbolic shield against the terror of death” (p. 20), whereas the loss or disruption of such relationships may be associated with heightened death awareness. Supporting this perspective, Rehnsfeldt and Arman [85] found that close relationships, including romantic partnerships, were associated with existential health following the trauma of a tsunami disaster. Prasinos and Tittler [80] underscored the link between romantic love and existential dimensions, identifying a significant association between love styles and existential meaning. Notably, the agape love style (characterized by altruistic, unconditional love without expectation of reciprocity) was positively associated with a sense of meaning in life. Conversely, the erotic love style, which prioritizes physical attraction and sexual appeal, and the pragmatic love style, which is guided by practicality and strategic considerations, demonstrated only weak associations with existential meaning.

Considering these perspectives and empirical findings, romantic love may not only be linked to individuals' existential experiences but also associated with their existential orientations. It may therefore be informative to examine the association between problematic engagement with online dating apps and pornography and the existential sense of love, as these platforms and the experiences they facilitate may be linked to attitudes toward romantic love and romantic relationships.

### Could problematic engagement with online dating apps and pornography be linked to a weaker perceived existential meaningfulness of love?

A review of the literature on problematic engagement with online dating apps and pornography suggests that these behaviors may be associated with the extent to which individuals attribute meaning and existential depth to romantic love and romantic relationships.

Examining the experiences of online dating app users, Kacprzak-Wachniew et al. [61] identified a "hurt" cluster, comprising users who frequently encounter distressing experiences, such as receiving unsolicited sexual content, being pressured into sexual encounters, experiencing deception, and facing disappointment in real-life meetings. Through interviews with problematic online dating app users, Lash [67] found that many users tend to “treat fellow dating application users as lesser than individuals they might meet organically.” In a longitudinal study, Sharabi et al. [94] found that online dating app users increasingly experience burnout, characterized by emotional exhaustion and a sense of inefficacy. Additionally, Arraichi and Ghourdou [8] identified shallow interactions (i.e., a lack of meaningful connections and genuine conversations) and unrealistic expectations as primary sources of dissatisfaction among online dating app users. Similarly, in an interview-based study, Lisa [68] found that many participants regarded online dating apps as unsuitable for developing romantic relationships, citing a lack of authenticity, superficial conversations, and minimal personal investment in building genuine connections.

Likewise, research on the lived experiences of problematic pornography consumers suggests potential associated love-related existential consequences. Palazzolo and Bettman [76] found that many individuals with problematic pornography use reported difficulties in establishing and maintaining intimacy, feelings of alienation (i.e., detachment from others), and distorted attitudes and unrealistic expectations regarding romantic relationships (e.g., objectification of partners, difficulty reconciling idealized images of a partner with reality). Ince et al. [56] similarly found that problematic pornography users often experience internal conflicts (e.g., discomfort regarding previously viewed content, which was frequently described as sexually demeaning, disturbing, or unethical), altered patterns of sexual arousal, shifts in sexual preferences and intensity (e.g., frequent content-switching, seeking increasingly novel or extreme material), negative emotional and cognitive experiences (e.g., anhedonia, mental exhaustion), and disruptions in their sex lives (e.g., diminished connection with real-life partners, unrealistic expectations about real-life sex and relationships, decreased desire for a real-life partner, and reduced enjoyment of orgasm with a real partner). Hanseder and Dantas [48] also reported that many problematic pornography users in their study demonstrated an escalating consumption of extreme content (e.g., hardcore videos). Moreover, empirical evidence suggests that pornography consumption is linked to behaviors that may indirectly reflect a diminished belief in the meaningfulness of romantic love, such as greater engagement in infidelity (see [70] for a review), more favorable evaluations of non-monogamous relationships [31], and participation in group sex [46].

Taking into account the experiences encountered during online dating app use and pornography engagement, such use, particularly when compulsive, may be linked to how users attribute meaning to romantic love, as personal experiences are associated with the formation and modification of one's perceptions and sense of meaning. *Meaning-making* is a fundamental aspect of human life. In response to life experiences, particularly stressful ones, individuals appraise the meaning of these experiences and adjust their perspectives accordingly. This process involves seeking comprehensibility, assimilating new information, and accommodating changes, all of which contribute to both the attributed meaning of specific experiences and broader meaning-making approaches (see the *Integrated Model of Meaning-Making*; [77]). A central dimension of meaning-making is *existential*, wherein individuals strive to make sense of their experiences from an existential perspective [65]. Gustavsson [45] discussed the concept of *existential configurations*, suggesting that individuals interpret their experiences through existential themes, and that personal, social, and cultural frameworks are associated with the shaping of these meanings. To date, various studies have explored experiences through the lens of existential meaning-making. For instance, Ueland (2019) examined existential experiences related to living with obesity, finding that some individuals may perceive obesity as an existential burden, experiencing objectification and alienation as human beings. Prinds et al. [81] investigated existential meanings in the transition to motherhood. Moreover, several studies have explored existential meaning adjustments in response to health challenges, such as receiving a diagnosis of serious illness (e.g., [51]). Meanwhile, existential meaning-making processes can also extend to love and family development experiences. These may include existential reflections on identity and intimacy in relationships, the value of romantic partnerships, marriage, infertility, miscarriage, and parenting [7].

Considering that individuals with problematic engagement with online dating apps and pornography may encounter negative and significant experiences, as documented in existing lived-experience studies (e.g., engaging in shallow interactions on dating apps or exposure to extreme pornographic content; [48, 68]), and given that existential meaning-making appears to play a role in how individuals understand and evaluate romantic love and relationships [7], an important question arises: *Could problematic engagement with dating apps and pornography be associated with existential perspectives on romantic love?*

### The current study

The current study focuses on problematic engagement (versus frequency of use) with online dating apps and pornography [15, 16, 43]. Problematic use of these platforms refers to addiction-like engagement characterized by salience (preoccupation and increasing craving for engagement), mood modification (engaging as a coping strategy to improve mood), tolerance (the need for increased engagement over time), withdrawal (unpleasant responses to the cessation or reduction of engagement), conflict (experiencing conflicts in everyday life due to engagement), and relapse (the tendency to revert to engagement after abstinence; see [44]).

The current study surveyed Iranian users of online dating apps and pornography websites. Recent research has highlighted the intensity of passionate love with existential depth, as well as agape love (i.e., unconditional love with a tendency toward self-sacrifice), among Iranians in romantic relationships (e.g., "An existence without my loved one would be dark and dismal"; see [86], p. 149). This may render the population particularly suitable for examining how problematic engagement with online dating apps and pornography is linked to attitudes toward the existential meaningfulness of romantic love.

Despite certain characteristics that may complicate research in Iran in the areas of online dating apps and pornography, such as government filtering of many dating apps and all sexual content websites (requiring the use of a VPN for access) and the presence of sociocultural and religious factors (e.g., prohibition of engagement with dating apps and pornographic content under the current religious governance; see [38, 39]), emerging trends suggest that findings from this population are not necessarily limited in generalizability. Recent studies highlight two trends in Iranian society: first, dating and mating strategies in Iran have undergone considerable modernization over the past decade, with many individuals, particularly among the younger generation, actively using dating apps [63]; and second, pornography use and the prevalence of problematic engagement in Iran appear to be comparable to those observed in other countries [79]. Given these recent shifts, while the distinctive characteristics of this population (e.g., restricted accessibility to dating apps and pornographic websites) should be taken into consideration, the findings are expected to exhibit a degree of generalizability to other societies. This study is not conceptualized as culturally specific and does not aim to provide culturally specific conclusions for the Iranian population; rather, it seeks to offer generalizable results, though the specific characteristics of the current sample should be considered when interpreting the findings.

Along with the primary objective of this study, which is to examine the association between problematic engagement with online dating apps and pornography and the existential meaningfulness of romantic love, the present study also provides preliminary findings on the association between mindful use of social media, defined as mindful awareness during social media engagement (e.g., with low automaticity; see [90–92]), and problematic online dating app use and problematic pornography use. Recent discussions suggest that mindful use of social media may play a protective role in relation to the life routines and health of individuals. These findings may offer initial insights into whether greater mindful awareness during social media use is associated with lower tendencies toward problematic engagement with online dating apps and pornography. If so, this may suggest that individuals who use social media mindfully may extend this mindful approach to other forms of media consumption.

## Methods

### Participants

The study included 664 Iranian adult users of online dating apps and pornography websites (359 female, 305 male; age range = 18–60 years; *M*_*age*_ = 26.42, *SD*_*age*_ = 7.59). Inclusion criteria required participants to have an active account and usage of at least one dating app in the past year, as well as consuming pornography for a minimum of 30 min per week over the past month. Of the participants, 6% had completed primary school education, 30.9% had completed secondary school, and 63.1% held a university diploma. Regarding sexual orientation, 89.9% self-identified as heterosexual, 0.5% as homosexual, 6.8% as bisexual, and 2.8% identified with other sexualities. A total of 58.7% self-reported being single, while 41.3% of participants reported being in a relationship. 28.8% identified Iranian dating apps as their primary platform, followed by Badoo (20.8%), Bumble (15.1%), OkCupid (8.9%), and Tinder (8.9%). 17.5% of participants reported using other apps (e.g., Hinge, eHarmony) as their primary dating app.

Ethical approval for this study was obtained from the Institutional Review Board of Pázmány Péter Catholic University (No. 2024/33). All procedures adhered to the National Statement on Ethical Conduct in Human Research, the Declaration of Helsinki of the World Medical Association, and the Ethics Code of the American Psychological Association. Informed consent was obtained from all participants prior to enrollment.

### Measures

The *Problematic Online Dating Apps Use Scale* (*PODAUS*; [43]) was used to assess problematic engagement with online dating apps, based on the Addiction Components Model [44]. The PODAUS is unidimensional and consists of six items (e.g., “I become restless if I am unable to use dating apps.”, “I have tried to cut down my dating app use but I have been unable to do it.”). Respondents rated their agreement using a Likert scale, ranging from *Strongly disagree* (1) to *Strongly agree* (5). Higher scores indicate a higher level of problematic use of online dating apps. The reliability of the PODAUS in the current study was good (*α* = 0.89).

The *Short Problematic Pornography Consumption Scale* (*PPCS-6*; [15, 16]) was used to assess problematic pornography consumption with six items. An example item is: “I neglected other leisure activities as a result of watching porn.” Respondents were instructed to consider their experiences over the past six months and rate each item using a 7-point Likert scale ranging from *Never* (1) to *All the time* (7). The PPCS-6 demonstrated good internal consistency in the current study (*α* = 0.88).

Inspired by the *Scale for Existential Thinking* (*SET*) and the construct of existential thinking [4], a single item was developed to assess the extent to which respondents engage with the concept of love from an existential perspective. The item was phrased as follows: “Have you ever spent time reading, contemplating, or discussing the philosophy of love? For instance, exploring questions such as: What exactly does love mean? Is love an illusion or a lasting truth? What is the purpose of love? Can love provide meaning and significance to life?” In line with the SET, the response options utilized a Likert scale ranging from *No or rarely* (1) to *All the time* (5), with an additional option, *I don’t know* (6). Responses marked as *I don’t know* were treated as missing data. No participants in the current study selected this response.

Drawing on established measures of meaningfulness (e.g., [49]), meaningful engagement in activities (e.g., *Engagement in Meaningful Activities Survey*; [40]), and domain-specific meaningfulness (e.g., meaning in work; [19]), a single item was developed to evaluate the extent to which respondents attribute meaningfulness to love. The item stated: “Love is meaningful to me; it imparts a sense of meaning to my life and existence.” Responses were recorded on a 5-point Likert scale ranging from *Strongly disagree* (1) to *Strongly agree* (5), with higher scores reflecting a greater perception of love as a source of meaning.

In this study, two measures of existential reflection on love and perceived meaningfulness of love were developed and were single-item measures. Growing evidence supports the applicability of single-item measures and their comparable functioning to multi-item measures (e.g., [5, 103]). Considering that the constructs of existential reflection on love and perceived meaningfulness of love are holistic (global/generic) constructs, single-item measures can be considered reliable, as research suggests that single-item measures are appropriate for such constructs [5, 30, 87]. In this study, the two measures aimed to capture the targeted constructs holistically and generically, similar to single-item measures of psychological well-being that ask participants to holistically rate their overall life satisfaction. Moreover, the content validity of the two measures was evaluated by six subject matter experts, who rated the relevance of each measure to the targeted constructs on a 4-point scale (1 = *not relevant*, 4 = *highly relevant*). The item-level content validity index for the measure of existential reflection on love was 0.83 and for perceived meaningfulness of love was 0.83, both of which exceed the recommended threshold of 0.78 [78].

The *Subscale of Pragmatic Features of Being in Love* of the Measure of Perception and Understanding of Love in Jankowiak et al.’s study [57] was used. This measure includes statements reflecting both idealized aspects (15 items; e.g., “I will do anything for my lover”) and pragmatic aspects (9 items; e.g., “Love makes fools of us all”, “Love dies without money”) of love. It represents an enhanced version of the scales originally developed by De Munck et al. [28, 29] to assess positive and negative perceptions of love, with additional items that cover aspects such as the perceived durability of love and the economic foundation of love. While Jankowiak et al. [57] employed a forced-choice response format (Agree or Disagree), the present study, in line with De Munck et al.’s [28] approach, utilized a 4-point Likert scale, ranging from *Completely disagree* (1) to *Completely agree* (4). Higher scores reflect a stronger belief in the pragmatic aspects of love. The scale demonstrated acceptable internal consistency in this study (*α* = 0.71).

The *Emotional Promiscuity Scale* (*EPS*; [60]) was used to assess emophilia. This scale comprises ten items (e.g., “I fall in love easily”, “I tend to jump into relationships”) that evaluate the tendency to develop romantic feelings quickly, frequently, and with less selectivity. The response options for the first nine items range from *Strongly disagree* (1) to *Strongly agree* (5), while the final item is rated from *None* (1) to *4 or more* (5). Although the items assess both the ease and frequency with which one develops romantic feelings, these two dimensions are highly correlated, and it is therefore recommended to combine them into a single measure (see [88]). The EPS demonstrated acceptable internal consistency (*α* = 0.64).

The *Mindful Use of Social Media Scale* (*MUSMS*; [90, 92]) was used to assess participants’ mindful awareness during social media use. This unidimensional scale includes seven items, such as, “It seems I am running on automatic during social media use, without much awareness of what I am doing in social media.” Respondents rate each item on a 6-point Likert scale ranging from *Almost never* (1) to *Almost always* (6). All items are reverse-scored, with higher total scores reflecting greater mindful engagement with social media. In the present study, the MUSMS demonstrated good internal consistency (*α* = 0.87).

### Procedure and data analysis

The survey of this study was advertised in a number of Iranian Psychology social media channels. The survey was administered in Persian and via Google Form. Completion of all survey items was mandatory, and there was no missing response in the recorded datasets.

For data analysis, Structural Equation Modeling (SEM) was employed to investigate the associations between problematic online dating app use and problematic pornography consumption (as latent predictors) with existential reflection on love, perceived meaningfulness of love (as single-item observed outcome variables), belief in the pragmatism aspect of love, and emotional promiscuity (as latent outcome variables). The effects of age, gender (i.e., female and male), education (i.e., primary or secondary school education, and university diploma), relationship status (i.e., single and partnered), and sexual orientations (i.e., heterosexual and non-heterosexual) were taken into account in the analysis (as observed control variables). The mean- and variance-adjusted weighted least-squares estimator (WLSMV) was employed, which is appropriate for categorical indicators [33] and was suggested for SEM models including the EPS and the PODAUS. Second, MUSMS was added to the model as a latent predictor of PODAUS and PPCS-6. In this analysis, direct paths between the MUSMS and the existential love outcome variables were not specified, based on the focus of the present study and relevant theoretical considerations. The widely-used, recommended fit indices were considered for model fit evaluations [12, 21]: Comparative Fit Index (CFI; ≥ 0.95 for good, ≥ 0.90 for acceptable), Tucker-Lewis Index (TLI; ≥ 0.95 for good, ≥ 0.90 for acceptable), and the Root-Mean-Square Error of Approximation (RMSEA; ≤ 0.06 for good, ≤ 0.08 for acceptable) with 90% confidence interval (CI). Based on the modification indices, error covariances were added to the models between the 2nd and 8th, and the 4th and 5th items of the Subscale of Pragmatic Features of Being in Love, and between the 6th and 7th, and the 4th and 5th item of the EPS. Error covariances were added to improve model fit. The similar wording and phrasing of the items render these error covariances interpretatively justifiable (e.g., “Romance without money is not good.” [item 4] and “Romantic love needs strong economic support.” [item 5] in the Subscale of Pragmatic Features of Being in Love, which are conceptually and semantically overlapping). Data analysis was conducted using IBM SPSS Statistics 21.0 and Mplus 7.4 [73].

## Results

**Fig. 1:**
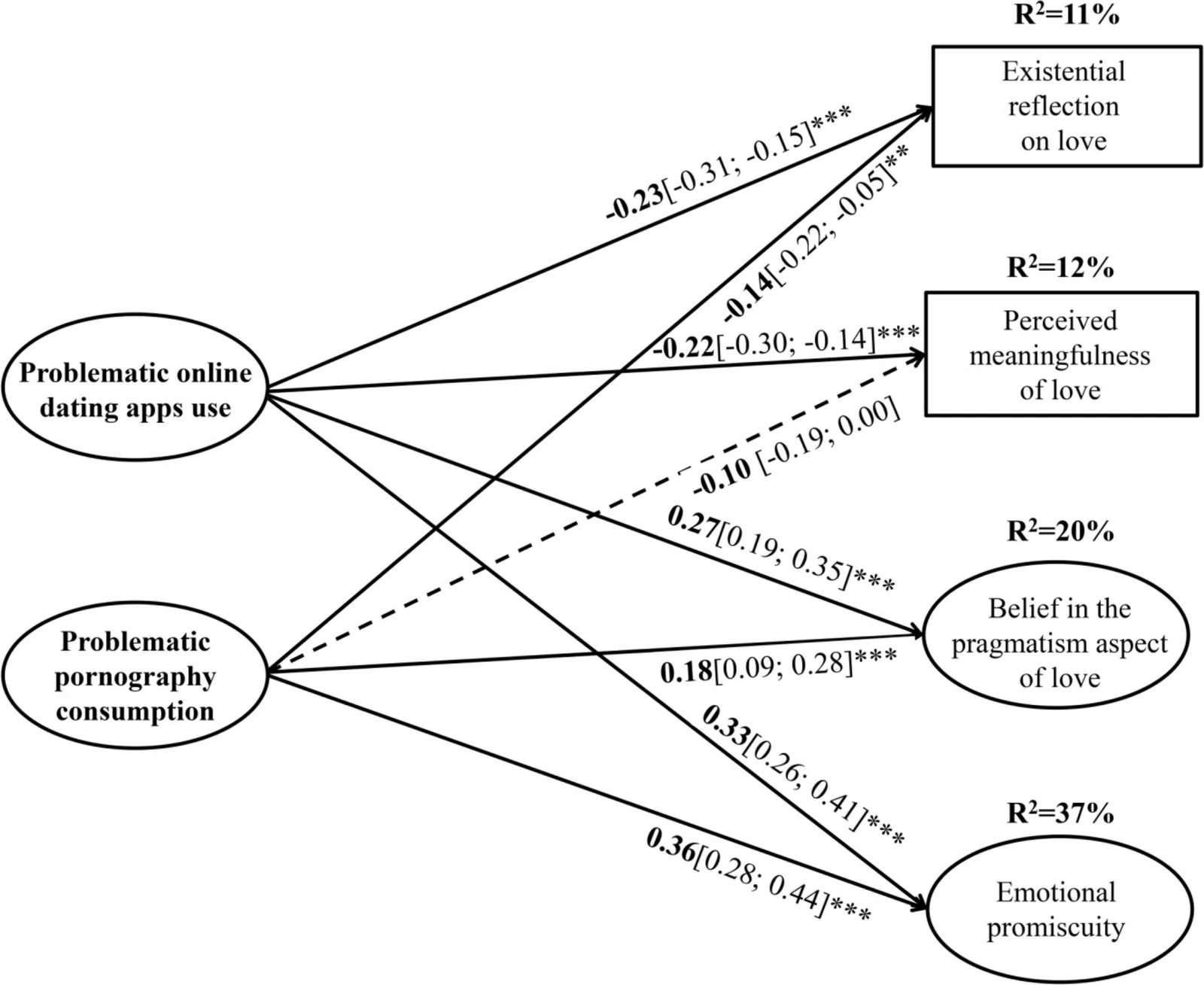
SEM representing the associations between problematic online dating app use and problematic pornography consumption and existential perceptions of love. *Notes*. ***p < 0.001; ** *p* < 0.01. Dashed arrow represents a nonsignificant path. Standardized beta regression coefficients are presented on the arrows with 95% confidence intervals in brackets. Ovals represent latent variables, whereas rectangles represent single-item observed variables. Gender, education, relationship status, and sexual orientation were included in the model as control variables

First, SEM was conducted to examine the association between problematic online dating app use and problematic pornography consumption and existential perceptions of love (see Fig. 1). The model fit was satisfactory (*χ*^*2*^ = 2,153.315, *df* = 613, *p* < 0.001; *CFI* = 0.917, *TLI* = 0.906, *RMSEA* = 0.062 [90% CI = 0.059–0.064]). Both problematic online dating app use and problematic pornography consumption were negatively associated with existential reflection on love (*β *_*Problematic online dating app use*_ = −0.23, *p* < 0.001; *β *_*Problematic pornography consumption*_ = −0.14, *p* < 0.01) and positively associated with belief in the pragmatism aspect of love (*β *_*Problematic online dating app use*_ = 0.27, *p* < 0.001; *β *_*Problematic pornography consumption*_ = 0.18, *p* < 0.001). Moreover, both problematic online dating app use (*β* = 0.33, *p* < 0.001) and problematic pornography consumption (*β* = 0.36, *p* < 0.001) were positively associated with emotional promiscuity. Based on the overlapping 95% CIs (see Fig. 1), no significant difference in the strength of these associations were found across problematic online dating app use and problematic pornography consumption. However, problematic online dating app use was negatively associated with the perceived meaningfulness of love (*β* = −0.22, *p* < 0.001), while the corresponding association with problematic pornography consumption was non-significant.

Problematic online dating app use and problematic pornography use were positively correlated (*r* = 0.36, *p* < 0.001). Overall, the largest proportion of variance explained by the predictors was found in emotional promiscuity (37%), while the explanatory power of the problematic behavior variables together with the control variables was modest (11–20%) for all other existential outcome variables.

**Fig. 2:**
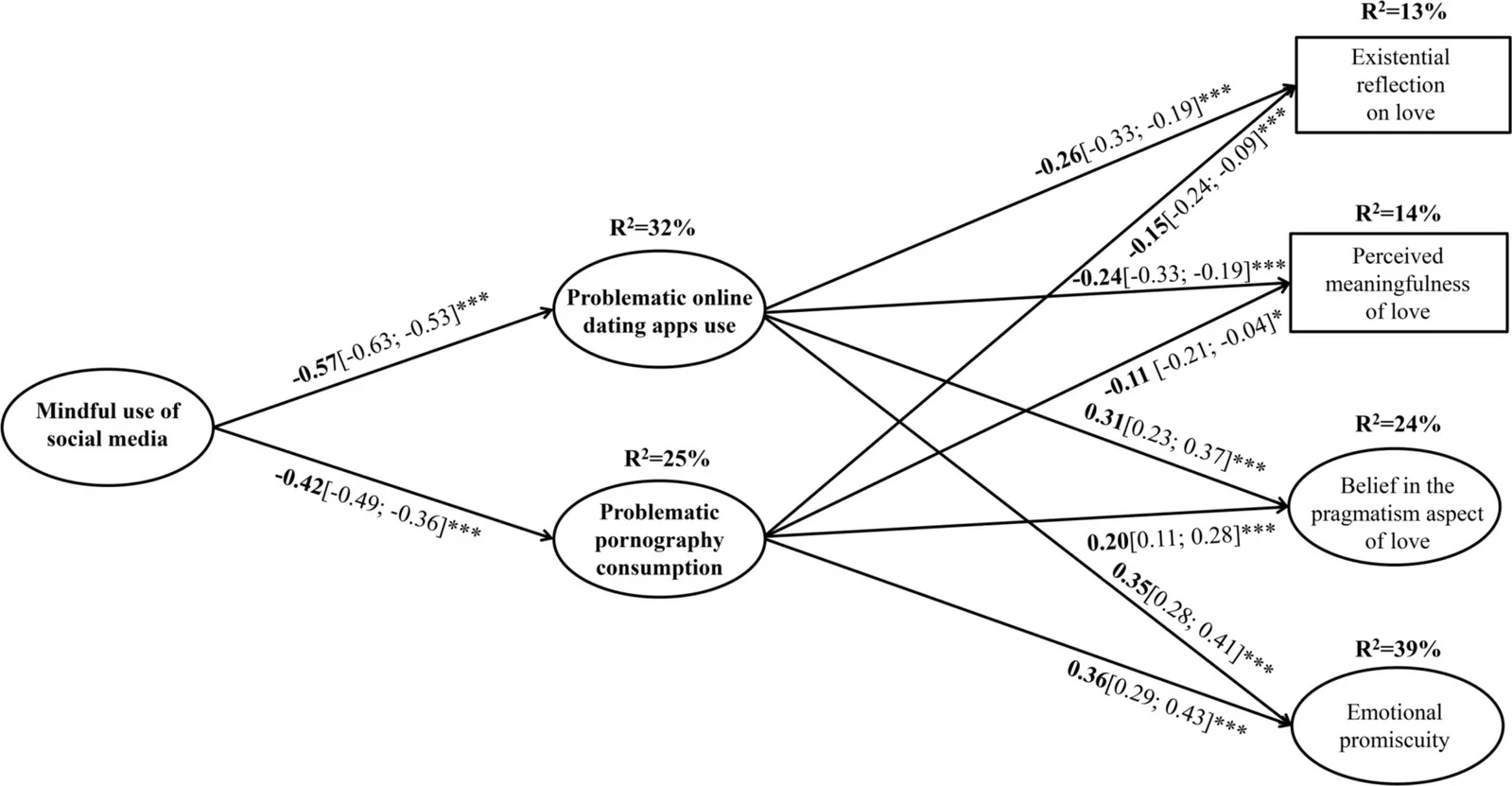
SEM representing associations between mindful use of social media, problematic online dating app use, problematic pornography consumption, and existential perceptions of love. *Notes.* ***p < 0.001; ** *p* < 0.01. Standardized beta regression coefficients are presented on the arrows with 95% confidence intervals in brackets. Ovals represent latent variables, whereas rectangles represent single-item observed variables. Gender, education, relationship status, and sexual orientation were included in the model as control variables

Second, mindful use of social media was added to the model to examine how it is associated with problematic online dating app use and problematic pornography consumption (see Fig. 2). The model fit was satisfactory (*χ*^*2*^ = 2,736.713, *df* = 886, *p* < 0.001; *CFI* = 0.920, *TLI* = 0.912, *RMSEA* = 0.056 [90% CI = 0.054–0.058]). Mindful use of social media was negatively associated with both problematic online dating app use (*β* = −0.57, *p* < 0.001) and problematic pornography consumption (*β* = −0.42, *p* < 0.001). However, the association was stronger for problematic online dating app use, as indicated by non-overlapping confidence intervals. Additionally, the explanatory power of mindful use of social media with control variables was slightly higher for problematic online dating app use than for problematic pornography consumption. The strength of associations between the two problematic behaviors and existential perceptions of love did not change significantly, except for the association between problematic pornography consumption and perceived meaningfulness of love, which became significant. Again, no significant difference could be found in the strength of associations with existential outcomes across the two problematic behaviors. Problematic online dating app use and problematic pornography use were positively correlated (*r* = 0.13, *p* < 0.001).

## Discussion

Beyond the psycho-socio-sexual outcomes thus far associated with problematic online dating app use and problematic pornography consumption, an important question remains: Might these problematic behaviors also be linked to existential romantic outcomes, such as a weak belief in romantic love as existentially meaningful, post-materialist, and worthy of fidelity? This study aimed to provide preliminary empirical evidence on the relationships among problematic online dating app use, problematic pornography consumption, and existential perceptions of romantic love, while also exploring, as a secondary goal, the association between mindful social media use and problematic engagement with dating apps and pornography.

The results showed associations of problematic online dating app use and all measures of existential perspectives on romantic love examined in this study. Specifically, higher levels of problematic online dating app use were associated with lower existential reflection on love, lower perceived meaningfulness of love, a stronger belief in the pragmatism aspect of love, and greater emotional promiscuity. These results may be interpreted in light of the likelihood of negative experiences associated with dating app use, as well as the unrealistic and distorted perceptions that such platforms may, to some extent, foster among users, particularly when engagement becomes compulsive (i.e., addiction-like).

Online dating app users may encounter distressing experiences on these platforms, such as deception, disappointment in online and in-person interactions [61], exposure to excessively high standards of partner selection, and inauthentic communication [8, 94]. Such recurrent experiences may gradually erode an existentially meaningful conception of romantic love (e.g., viewing love and one's romantic partner as profoundly significant and worthy of personal sacrifice) and instead foster a more pragmatic and transient approach to romantic connections, wherein love and romantic partners come to be seen as undeserving of existential investment and sacrifice. Experiencing repeated short-term and unfulfilling interactions, coupled with frequent rejection based on superficial appearance and material evaluations, on online dating apps may, to some extent, make it difficult for some users to construct a coherent and meaningful narrative about romantic love. When, on dating apps, individuals are rapidly categorized as favorable (swiped right) or unfavorable (swiped left) based on a few images and short bios within seconds, and when post-match interactions sometimes revolve around superficial and materialistic topics (e.g., occupation, income, place of residence) and tend to be fleeting or incomplete (see *Marketized Love*; [11]), some users, particularly compulsive users who likely experience these interactions more intensely, may gradually shift in their perceptions of romantic love and relationships. Some users may come to view romantic relationships as lacking depth and unworthy of existential investment.

Moreover, the availability of hundreds of potential matches on online dating apps may foster, in some cases, the perception that any given relationship or match lacks meaningful significance, as some users may come to think of themselves as merely one among many options, and of their matches as equally interchangeable. This perspective may, in some cases, be associated with the assumption that any current relationship is merely one of many possibilities, easily replaceable, and ultimately lacking existential meaning and purpose [93, 99]. Furthermore, a substantial proportion of online dating app users seem to seek short-term, casual encounters rather than meaningful relationships [47]. Such an environment may, in some cases, be associated with a weakening of existential views on romantic love among some users, particularly compulsive users, such as a greater acceptance of pragmatic and emotionally transient romantic relationships, possibly as a way to align with prevailing app-based dating culture and reduce vulnerability to experiences such as rejection. Therefore, considering empirical evidence on the negative experiences of some users on dating apps (e.g., [8, 61, 67, 94]), problematic dating app use is assumed to be associated with a weakened existential valuation of romantic love and relationships (see *Media Effects Theories* and *Existential Meaning Configuration*; [45, 65, 101]), particularly among problematic users with heightened exposure to negative dating experiences, such as superficial conversations, fleeting relationships, and evaluations based primarily on materialistic or appearance-based criteria.

Regarding problematic pornography consumption, the results showed a similar pattern, with associations observed between higher levels of problematic pornography consumption and lower existential reflection on love, a stronger belief in the pragmatic aspect of love, and greater emotional promiscuity. Although the relationship between problematic pornography consumption and perceived meaningfulness of love was statistically non-significant in the first model, the association became significant in the second model. The dominant themes in pornographic content may help contextualize these results. Pornography primarily emphasizes sexual arousal and pleasure while placing less focus on intimacy and romantic relationships [56, 76]. Such content may influence how some users perceive romantic love and what they expect from romantic relationships, potentially reflected in a greater emphasis on sexual passion and a reduced prioritization of intimacy (see *Media Effects Theories* and *Mediated Construction of Reality Theory*; [27, 101]). Moreover, problematic engagement with pornography may increase the likelihood of exposure, in some users, to increasingly extreme sexual content (e.g., hardcore content) that extends beyond what is typically associated with romantic relationships. Research suggests that, for some users, pornography consumption may escalate toward more extreme content over time [48]. This progression may, in some users, weaken the perception of love as meaningful, exclusive, and worthy of fidelity. Indeed, the expansion of sexual interests and desires through problematic pornography exposure may, in some cases, create the impression that meaningful, committed relationships impose constraints on personal gratification. Therefore, problematic pornography consumption may, to some extent, weaken the existential valuation of romantic love (see *Existential Meaning Configuration*; [45, 65]) and contribute to the assumption that romantic love lacks depth, priority, and worthiness of significant existential investment. Meanwhile, based on the present results, problematic pornography consumption (a non-interactive and parasocial experience) appears to be less strongly associated with the perceived existential meaningfulness of love compared to problematic dating app use (an interactive experience).

As expected, the results showed a positive association between problematic online dating app use and problematic pornography consumption. This finding aligns with prior research demonstrating that different forms of problematic media use, and, more broadly, behavioral addictions, often appear to be interconnected [42, 43]. It appears that the more problematic a user's engagement with dating apps or pornography, the more problematic their engagement with the other. This is consistent with the assumption that many addictions can be comorbid and that engagement with one form of addictive behavior may heighten the risk of transitioning to another [107].

Interestingly, mindful social media use was negatively associated with both problematic online dating app use and problematic pornography consumption. These results suggest two possibilities. First, social media use may increase the tendency to engage with dating apps and sexual content. For instance, advertisements promoting dating app downloads, posts about dating (e.g., clips of blind dates), and sexually suggestive content may contribute to this pattern. Problematic social media use may heighten the desire to use dating apps and consume pornography, particularly as these behaviors may be perceived as integral to contemporary media culture. To align with prevailing media consumption norms and avoid feelings of missing out, some users may feel compelled to engage with these platforms to some extent. Mindful social media use, by potentially reducing impressionability to social media content [90–92], may help disrupt this pattern. Second, given that problematic use of different media can be interconnected and comorbid (see [42, 107]), it is possible that mindful engagement with one type of media platform (e.g., social media) may extend to other media-related behaviors (e.g., mindful dating app use) or even non-media behaviors (e.g., mindful eating). In other words, there may be an interconnected web of mindful engagement across different media platforms, such that individuals who engage mindfully with one form of media may be more likely to extend this mindful approach to other platforms. Future research could explore the role of mindful awareness during dating app use and pornography consumption.

Inspired by the benefits documented in relation to mindful engagement with media and media content (e.g., mindful social media use; [90–92]), *mindful use of dating apps* and *mindful consumption of pornography* may help users regulate their engagement with these platforms, fostering a more balanced and autonomous pattern of use. This is particularly important as dating apps are increasingly designed to foster user dependency through new features, while pornography platforms continue to expand their content and present it in easily consumable, bite-sized formats to sustain user engagement. Currently, dating apps and pornography platforms lack protective measures for users, such as the one-hour usage notifications available on platforms like TikTok and Instagram. However, even if such measures were implemented in the future, their effectiveness would likely remain limited. Many of these interventions have been shown to be ineffective in curbing problematic behaviors among users, and some companies may feign concern for user well-being while making minimal genuine efforts to reduce harm (see *screenwashing*; [62]). Therefore, cultivating mindful engagement by users themselves with these platforms may serve as a more viable approach. Mindful awareness during dating app use and pornography consumption, that is, maintaining awareness of one's motivations, emotions, and thoughts while engaging with these platforms, is presumed to facilitate a more balanced and intentional pattern of use, and ultimately support users' well-being. Future research is encouraged to further explore this avenue and provide empirical evidence.

This study proposed assumptions regarding how problematic engagement with online dating apps and pornography is associated with the attitudes toward existential meaningfulness of romantic love. However, these remain assumptions, as the current study focused on relationships rather than underlying processes. Future research could explore *how* problematic engagement with online dating apps and pornography is linked to weakened existential meaning in romantic love. Additionally, it is important to clarify that this study conceptualized and discussed the relationship from the perspective of problematic online dating app use and pornography consumption being associated with reduced existential meaningfulness in romantic love. However, an alternative direction, whereby lower existential meaningfulness of romantic love is associated with greater problematic engagement with dating apps and pornography, is also plausible. Furthermore, in line with the *Reinforcing Spirals Model* [96], it is possible that these factors influence each other simultaneously in a reciprocal manner. Future research employing advanced methodologies, such as experimental designs, is essential to establish more definitive conclusions regarding the direction of these effects. The present study adopts an interpretative approach aligned with the dominant perspective in existing research, focusing on the associations between online dating app and pornography use and their outcomes (e.g., interview studies on the gradual negative outcomes of problematic engagement with online dating apps and pornography; [56, 67, 68]).

This study is not without limitations. First, due to the cross-sectional nature of the data collection, we are unable to draw confident conclusions on causality and long-term effects. As discussed earlier, a reverse model may also be plausible. However, the present conceptual and interpretative direction was chosen in light of theoretical (e.g., theories on media effects; see [101]) and empirical evidence (e.g., lived experience studies documenting negative experiences of users during problematic engagement with online dating apps and pornography; [56, 68]) supporting this direction. Future research could benefit from longitudinal and experimental approaches. Second, this study was conducted on an Iranian sample. Considering the channels used for advertising and recruiting participants, which were social media channels used by Iranians inside the country rather than those of the diaspora, the sample reflects the charactristics of Iranians residing within Iran. Several characteristics of this sample warrant consideration, such as restricted accessibility to online dating apps and pornographic content, given that many dating apps are blocked and all pornographic websites are inaccessible without the use of a VPN, the influence of the current Islamic religious government, which prohibits the use of dating apps and pornographic content [38], and sociocultural norms around sexual modesty and shame within an Eastern collectivistic context (e.g., [39]). For example, due to sexual modesty or shame, Iranian participants may exhibit bias in self-reporting their engagement with online dating apps and pornography. Research suggests the possibility of social desirability bias in self-report measures of pornography consumption [84]. Future research should examine samples from diverse sociocultural contexts to empirically confirm the generalizability of this study's findings. However, despite its focus on Iranian users, the study's findings may not be restricted to Iranian or similar societies and cultures, as evidence suggests that Iranian users are comparable to other societies in terms of dating app and pornography consumption [63, 79], and that romantic existentialism is present across diverse cultures, as reflected in several Western philosophical and scientific discussions on the existential meaning of romantic love [23, 37, 71, 80, 95]. Third, in this study, female and male participants were examined together. Considering that female and male internet users may differ in their engagement with online dating apps and pornography (e.g., [59]), and that Iranian female users may face greater sociocultural pressures against using these platforms [9], gender-focused studies may provide further insights. It is worth noting, however, that the number of female and male participants in this study was approximately equal and gender was controlled for in the model. Fourth, despite a few variables controlled in this study (e.g., age and education), several relevant variables, such as religiosity, that may be associated with engagement with these platforms [34] were not measured or controlled. Future studies incorporating such variables (e.g., religiosity, sexual modesty) could add greater confidence to the findings. Fifth, we relied on self-assessment tools to measure our variables. However, due to social desirability and errors of judgement, not all answers might give a correct reflection of reality. Future research might use a data donation approach to obtain more reliable input on participants’ dating app use and pornographic consumption. Sixth, a few of the measures used in this study were single-item measures. While there is evidence confirming the applicability of single-item measures and their comparable functioning to multi-item measures (e.g., [5, 103]), future studies employing multi-item measures or measures with subscales could add greater confidence and more nuanced layers to the findings of this study. Seventh, the EPS showed modest internal consistency in this study, which should be considered in the interpretation of the findings and in future investigations. Finally, our measures of media consumption were rather broad, we did not take differences into account in types of dating app use and types of pornographic websites. Future research might further specify the findings by checking whether the paths develop differently depending on the type of platforms that are being used and their content. It is also worth noting here that the current study focused specifically on problematic engagement with online dating apps and pornography. Future studies examining frequency of engagement, rather than problematic engagement alone, could provide an additional layer of evidence beyond the framework of addiction-like engagement.

Despite these limitations, this study has some implications. Firstly, there are implications for health practitioners. The connection between these problematic online dating app use and pornographic content and negative existential perceptions of love has significant mental health implications. If users begin to view romantic relationships as mere transactions or based on shallow metrics, this could lead to feelings of emptiness, alienation, or dissatisfaction. Mental health professionals might need to incorporate the effects of digital engagement into their therapeutic strategies, particularly when addressing issues related to relationship fulfillment, self-identity, and existential health. Moreover, policymakers and digital platform developers might benefit from our findings. Given the results, these stakeholders might think about developing features and guidance during media consumption that encourage more meaningful, intentional engagement. For the dating apps for instance, checks might be included to avoid shadow profiles and people might get extra warnings to answer their messages to avoid meaningless and dead conversations. Pornographic website developers as well might feature content that emphasizes consent, mutual respect, and healthy emotional connections between partners. If certain content does not fit with these descriptions, disclaimers might be used to inform and educate consumers on the fictional character in the content.

Overall, the findings of this study indicate that problematic use of online dating apps and pornography is associated with a weak perceived meaningfulness of romantic love. Additionally, mindful use of social media demonstrated negative associations with these problematic behaviors. Problematic engagement with online dating apps and pornography may, in some cases, shatter the existential meaning of romantic love and relationships. Therefore, users are encouraged to adopt a more *mindful* approach when engaging with these platforms.

## Data Availability

The deidentified dataset used in this study is openly accessible at the Open Science Framework: https://osf.io/zk2qs/?view_only=12187309352c4a13a6d69c97ccf7f596
